# The Crowded Medical Schools

**Published:** 1920-05-15

**Authors:** 


### THE CROWDED MEDICAL SCHOOLS.

A few days ago *The Times* drew attention, under the title of "A note of warning," to the rush of students to enter medical schools all over the country. At the beginning of last year, with the war ended and a steady, if gradual, demobilisation of medical or intending medical students from the Services, this crowding at the gates was a hardly surprising occurrence, but, in comparison, the situation at the moment presents several essential points of difference.

Not only is there a "rush" to enter the medical profession, but the majority of schools, considering the accommodation and teaching staffs available, are rapidly approaching a state of absolute overcrowding, and although response to recent financial appeals and academic extensions may do something to counter-balance this tendency, it is doubtful, should such a state of affairs continue, if the expected benefit from our reformed educational arrangements will accrue either to the individual or to the profession as a whole.

Also, we feel that after such widespread press activity in booming the great future of health work in this country and the tremendous number of ramifications and corresponding appointments which may arise from the workings of the new Ministry, it is not at all unlikely that exaggerated notions of prosperity are leading all kinds, suitable and utterly unsuitable people, to attempt an entry into the most arduous and at the same time most difficult of professions. It would seem, as *The Times* says, that "It is generally felt that a great new epoch is beginning, and that in a golden future there will be work for all." This decision may, as far as public health work is concerned, be to some extent justified; but before it grows into a generally accepted conclusion that the best road to prosperity for the scientifically trained lies in a medical career, the facts should be very carefully considered as they really are.

To define, even vaguely, the ideal personal qualifications of the doctor, either male or female, is beyond the aim of this note; but at least it must be realised that relatively few of those met with in average college life even approximately possess them. Mere ability to pass and win honours in examinations is an advantageous attribute, but in medicine it is by no means a complete equipment. Also, given the good or even ideal personality, the path of either the physician or surgeon is no easy way to riches. A small percentage, as in every other profession, achieve both wealth and greatness, but they are few and far between, and though it is true that in most circumstances a doctor can make a living, we do not think that medicine's just claims in this direction are so great as some of the young men and women now crowding to the schools would seem to imagine.

Physical condition, too, is a consideration. There is an annual list of no inconsiderable dimensions representing those in a medical career who lose health and strength and enter old or even middle age as broken-down men. Any authentic list of occupational mortality and sickness will provide remarkable evidence of this fact, and, though we would not for one moment dissuade the considered admission and entry of the country's best youth into the schools of a magnificent profession, yet we think that for the institutions themselves, the quality of the training they provide, and the students' own future, it would be well if those directly or indirectly responsible for the present "rush" were to realise more thoroughly the true circumstances which exist.

